# Fire Prevention: Appliances, Separating Partitions, and Staircases

**Published:** 1912-11-30

**Authors:** 


### FIRE PREVENTION.

### Appliances, Separating Partitions, and Staircases.

ONE of the features of the twentieth century is the demand for fire-resisting construction and alternative means of escape from all places where numbers of persons are congregated. This demand has probably been accentuated by the disastrous fires which are fortunately not so numerous in the hospitals as in London workshops. The gradual evolution caused by the Housing Acts and Town Planning Act is helping to clear away too closely built on areas, where fire can do such serious damage both to life and property, and it is being generally realised that prevention is better than cure.

Fire-fighting appliances are now brought to a very high state of perfection, and probably none is more suitable for the institutional buildings than the $CO_2$ type of extincteur made by the British Fire Appliances Company, Ltd. The merit of such appliances l'es as much in the reduction of insurance premiums as in actual use, unless fire drill forms part of the training of each member of the staff. We have practically no building materials that are absolutely fire-proof, though some are strongly fire-resisting. Different materials act in different ways under excessive heat: some disintegrate, some twist, some buckle, and all expand in different ratios; so that the building in which a severe fire has taken place is generally considered only fit for demolit'on.

Recent legislation provides for covering of a certain thickness in fire-resisting materials to all structural steel work to preserve it from the action of fire. This is about as far as we can go, except that our buildings can be sub-divided into small compartments by fire-proof doors, thus localising fire as much as possible and preventing its spread. The modern hospital door should for aseptic reasons be flush panelled with only a V joint where the panels join the framing; and if made of hardwood, as is usual, we have in this an ideal fire-resisting, almost fire-proof, door; for the surface, if charred, acts efficiently as a protective coating to the body of the door, which is some 2 inches in thickness. The separating partitions in which the doors are placed naturally must also be of fire-resisting construction.

SLAB PARTITIONS.

The majority of the patent slab partitions now on the market will burn at a very high temperature, such as would obtain in a severe fire; except probably those made of clay, which seem to stand severe tests remarkably well, and are, therefore, the safest for partitions and the enclosing of lift wells. These details point to the desirability of providing sufficient means of escape from the building to the open air and, where practicable, to the ground level. Th's is particularly essential in the case of the ward blocks, for panic, even without fire, can do much severe damage to bedridden patients.

The ideal ward, which has its balcony on the south end, should have its access staircase at the north end. A staircase should have a minimum width of 3 feet 6 inches in the clear, and there should be no steps whatever on the half landing, so that an easy turn is poss'ble. In the escape from the south balcony an inclined plane leading to the ground will be the ideal to strive for, but will often be found impossible, owing to restricted space and means of supervision. It is also of the utmost importance to have per'odical fire drill, so that each member of the staff is conversant with the means of preventing spread of fire and with the handling of those under charge, for occasional practice tends to give, more than any other method, that cool and calm deliberation if panic is to be avoided.

